# Aortic aneurysms: current pathogenesis and therapeutic targets

**DOI:** 10.1038/s12276-023-01130-w

**Published:** 2023-12-01

**Authors:** Min Ji Cho, Mi-Ran Lee, Jong-Gil Park

**Affiliations:** 1 Biotherapeutics Translational Research Center, Korea Research Institute of Bioscience and Biotechnology (KRIBB), 125 Gwahak-ro, Yuseong-gu, Daejeon, 34141 Republic of Korea; 2 Department of Biomedical Laboratory Science, Jungwon University, 85 Munmu-ro, Goesan-eup, Goesan-gun, Chungbuk 28024 Republic of Korea; 3 Department of Bioscience, KRIBB School of Bioscience, Korea University of Science and Technology (UST), 125 Gwahak-ro, Yuseong-gu, Daejeon, 34141 Republic of Korea

**Keywords:** Aneurysm, Aneurysm

## Abstract

Aortic aneurysm is a chronic disease characterized by localized expansion of the aorta, including the ascending aorta, arch, descending aorta, and abdominal aorta. Although aortic aneurysms are generally asymptomatic, they can threaten human health by sudden death due to aortic rupture. Aortic aneurysms are estimated to lead to 150,000 ~ 200,000 deaths per year worldwide. Currently, there are no effective drugs to prevent the growth or rupture of aortic aneurysms; surgical repair or endovascular repair is the only option for treating this condition. The pathogenic mechanisms and therapeutic targets for aortic aneurysms have been examined over the past decade; however, there are unknown pathogenic mechanisms involved in cellular heterogeneity and plasticity, the complexity of the transforming growth factor-β signaling pathway, inflammation, cell death, intramural neovascularization, and intercellular communication. This review summarizes the latest research findings and current pathogenic mechanisms of aortic aneurysms, which may enhance our understanding of aortic aneurysms.

## New Insights into Complex Pathologies of Aortic Aneurysms

Aortic aneurysms, a chronic aortic disease, can lead to life-threatening consequences. Recent advancements in understanding the pathophysiological mechanisms of aortic aneurysms have focused on the source of therapeutic targets. The heterogeneity of aortic cells during the progress of an aortic aneurysm is augmented by the alteration of gene expression that changes the phenotype and function of the aortic cells. Inflammatory cells, cytokines production, matrix metalloproteinases, smooth muscle cells phenotypic switching, SMCs death, neovascularization, and thrombosis contribute to the development and progression of aortic aneurysms. Neutralization of transforming growth factor-β in mouse aortic aneurysm models exhibits different consequences in the progress of aortic aneurysms depending on experimental designs. Extracellular vesicles contain various contents with biological activity and are released from donor cells to target cells or organs, potentially promoting or preventing the progress of aortic aneurysms.

This summary was initially drafted using artificial intelligence, then revised and fact-checked by the author.

## Introduction

Aortic aneurysm is a chronic aortic disease characterized by permanent localized dilatation of the aorta through adverse remodeling of the aortic wall, and it can subsequently progress to life-threatening consequences through aortic rupture, which has a mortality of over 80% and causes 150,000-200,000 deaths each year worldwide^1–3^. Aortic aneurysms are generally classified as thoracic aortic aneurysms (TAAs), which form in the ascending aorta, the arch, or the aorta above the diaphragm, or abdominal aortic aneurysms (AAAs), which are localized in the aorta below the diaphragm in the supra- or infrarenal regions^2^. Although distinct pathological mechanisms are present in TAA and AAA, many risk factors for aortic aneurysms are shared, including age, smoking, hypertension, hyperlipidemia, male sex, white race, and a positive family history^2,4–6^.

**Fig. 1:**
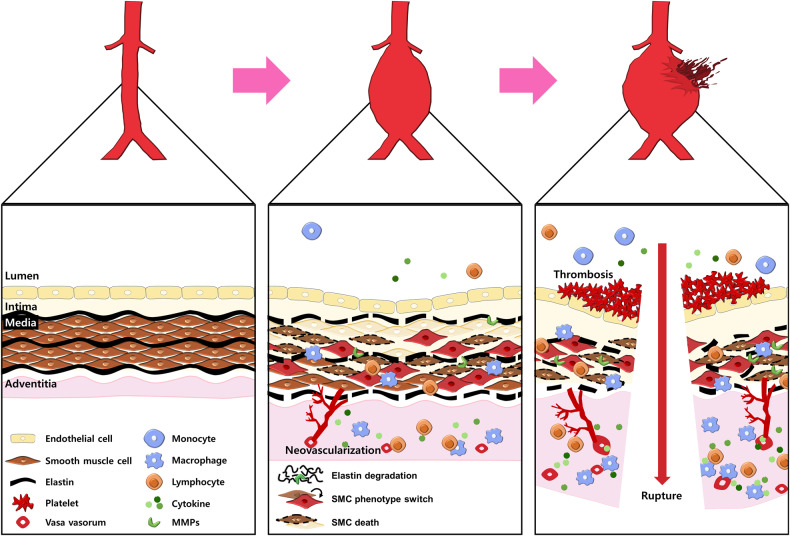
Pathogenesis of aortic aneurysms. Schematic diagrams showing events that contribute to the development and progression of aortic aneurysms from the healthy to the ruptured state. Aortic aneurysmal lesions are characterized by inflammatory cell infiltration, cytokine production, matrix metalloproteinase (MMP) activation, extracellular matrix (ECM) degradation, smooth muscle cell (SMC) phenotypic switching, SMC death, neovascularization, and thrombosis.

Aortic rupture is not only associated with increasing aneurysm diameters but also results from characteristic changes, which involve the progressive expansion and weakening of the three layers of the aorta: the intima, media, and adventitia^1,3^. Multiple pathological processes, including extracellular matrix (ECM) breakdown, inflammation, phenotype switching of vascular smooth muscle cells (SMCs), oxidative stress, and neovascularization, contribute to this process^7^. Furthermore, these biological mechanisms are thought to initiate the degradation of elastic fibers and alterations in collagen composition, ultimately compromising the structural integrity and reducing the flexibility of the aortic wall^1,2^. Although the pathological mechanisms of aortic aneurysms have been defined (Fig. 1), there are no effective drugs to treat aortic aneurysm growth or rupture^4^. In this review, we summarize recent advancements in aortic aneurysms and mainly focus on the pathophysiological mechanisms involving therapeutic targets.

## Heterogeneity and plasticity of cells in aortic walls during the progression of aneurysms

The aortic wall consists of multiple cell types that perform various functions to maintain homeostasis of the aorta^5^. Many types of cells, including endothelial cells (ECs), vascular SMCs, fibroblasts, pericytes, immune cells, and mesenchymal stem cells (MSCs), exist in the aortic wall, revealing the heterogeneity among these cell types^8,9^. The heterogeneity of aortic cells during the progression of an aortic aneurysm is augmented by alterations in the expression of genes that change the phenotype and function of aortic cells (Table 1). Control and aneurysm human aortic tissues reveal dynamic cell populations and differential gene expression^10^. Major wall cell types in the human ascending aorta were identified as 2 clusters of SMCs, fibroblasts, MSCs, ECs, monocytes/macrophages/dendritic cells (DCs), T lymphocytes, natural killer cells, mast cells, B lymphocytes, and plasma cells^10^. Multiple subtypes of SMCs, macrophages, and T lymphocytes revealed the diverse functions of these cells in the aortic wall^10^. In the aortic cells of mice, single-cell RNA sequencing identified 17 clusters with nine cell lineages of SMCs (4 clusters), fibroblasts (2 clusters), ECs (1 cluster), immune cells (5 clusters in monocytes and macrophages; 1 cluster in B lymphocytes; 1 cluster in T lymphocytes; 1 cluster in DCs), neural cells (1 cluster), and erythrocytes (1 cluster)^11^.

**Table 1:** The heterogeneity of aortic cells during the progression of aortic aneurysm.

Cell Type		Cell Marker	Tissue	Ref
Human				
SMC	contractile	ACTC1, ACTA2, MYL9, CARMN	Human ascending aortic tissue	^11^
	stressed	FOS, ATF3, JUN, HSPB8,		
	two proliferating	MGP, TPM4, MYH10		
	fibromyocyte cluster	ACTA2, MYL9, COL1A2, COL8A1		
Macrophage	M1-like	TNF, IL1B, NFKB1		^10^
	M2-like	MERTK, MRC1, STAB1, CD163		
Macrophage	Macrophage	CD14, FCGR3A, CD68, TFRC		^18^
Monocyte	Monocyte	CD14, FCGR3A, CD36, HLA-DRA		^18^
T lymphocyte	active CD4	CREM, CXCR6, RGCC, MR3C1, GZMB		^11^
	resting CD4	CCR7, IL7R, CCL20, KLRB1		
	regulatory CD4	IL2RA, CTLA4, TNFRSF18, ID3, LTB		
	active CD8	GZMK, CRTAM, CCL4, CMC1		
Endothelial cell	EC1 (high cell motility)	VWF, PECAM1, IFI27		^10^
	EC2 (high junction score)			
Fibroblast	Fibroblast1 (high elastin)	COL1A2, DCN, LUM, CLU, ELN		^10^
	Fibroblast2 (high fibrillin-1)	COL1A2, DCN, LUM, CLU, FBN1, LUM, DCN		
**Mouse**				
SMC	SMC	Acta2, My11, Mylk	Ang II and HFD-induced AAA mice models	^16^
SMC	quiescent-contractile	Myh11, Acta2, Tagln, Myl9	Elastase-treated AAA mice models	^11^
	proliferative-contractile	Fos, Jun, Klf2, Atf3, Dusp1		
	dedifferentiated	Ifrd1, Klf4, Atf3, Klf2, Ctss, Adamts1, Cxcl2, Ccl2, Mt1, Mt2, Hk2, Gata6		
	inflammatory-like	Ifrd1, Nrip2, Pln, Klf4, Atf3, Klf2, Ctss, Adamts1, Cxcl2, Ccl2, Sparcl1, Igfbp5, Sncg, Thbs1, Notch3		
Macrophage	Mo/Mφ_1-5	Cd14, Cd68, Adgre1, H2-Aa, Fcgr1		
	Mo/Mφ_3	Arg1, Egr2, Il1r2		
	Mo/Mφ_4	Ccl2, Ccl3, Cxcl10, Il1b, Mmp9, Ctsc		
Endothelial cell	EC1	Cdh5, Pecam1, Fabp4		
Fibroblast	Fibro_1 and _2	Dcn, Pdgfra, Col1a1, Col3a1		
Macrophage	Mφ-1 (anti-inflammatory)	Pf4, Mrc1	CaCl_2_-induced AAA mice models	^18^
	Mφ-2 (inflammatory)	Il1b, H2-Ab1		
	Mφ-3 (proliferation)	Mki67		

*SMC* smooth muscle cell, *Ang II* angiotensin II, *HFD* high-fat diet, *AAA* abdominal aortic aneurysm, *Mo/Mφ* monocyte/macrophage.

One of the most abundant cell types in the aortic wall is SMCs, which are responsible for vessel contraction. Various stresses, such as blood flow dynamics, oxidative stress, and inflammation, drive SMC phenotypic switching from a contractile to a proliferative/migratory and synthetic phenotype in aortic aneurysm^12–14^. Although contractile-to-synthetic phenotypic switching of SMCs has been suggested, recent single-cell transcriptomics and lineage tracing studies have revealed multiple subtypes of SMCs associated with disease progression in aortic aneurysms^10,11,15^. Mice with infrarenal abdominal aortic aneurysm were used to identify four subpopulations of SMCs: quiescent-contractile SMCs (*Myh11*^*+*^, *Acta2*^*+*^, *Tagln*^*+*^, *Myl9*^*+*^), proliferative-contractile SMCs (*Fos*^*+*^, *Jun*^*+*^, *Klf2*^*+*^, *Atf3*^*+*^, *Dusp1*^*+*^), dedifferentiated SMCs (*Ifrd1*^*+*^, *Klf4*^*+*^, *Atf3*^*+*^, *Klf2*^*+*^, *Ctss*^*+*^, *Adamts1*^*+*^, *Cxcl2*^*+*^, *Ccl2*^*+*^, *Mt1*^*+*^, *Mt2*^*+*^, *Hk2*^*+*^, *Gata6*^*+*^), and inflammatory SMCs (*Ifrd1*^*+*^, *Nrip2*^*+*^, *Pln*^*+*^, *Klf4*^*+*^, *Atf3*^*+*^, *Klf2*^*+*^, *Ctss*^*+*^, *Adamts1*^*+*^, *Cxcl2*^*+*^, *Ccl2*^*+*^, *Sparcl1*^*+*^, *Igfbp5*^*+*^, *Sncg*^*+*^, *Thbs1*^*+*^, *Notch3*^*+*^)^11^. Compared to the controls, only the inflammatory SMC subpopulation was increased during abdominal aortic aneurysm progression, whereas the other three SMC subpopulations were proportionally decreased^11^. Consistent with this, single-cell transcriptome analysis revealed 6 clusters of SMCs (*Acta2*^*+*^, *Myh11*^*+*^, *Mylk*^*+*^) in C57BL/6 J mice with or without a high-fat diet and an infusion of angiotensin II (Ang II), and after the challenge, one of the SMC clusters exhibited upregulated expression of genes involved in the reactive oxygen species (ROS) response, DNA damage response, inflammatory response, and cell death^16^. In human ascending aortic walls, 5 SMC or SMC-related clusters have been identified, including the contractile SMC cluster (*ACTC1*^*+*^, *ACTA2*^*+*^, *MYL9*^*+*^, *CARMN*^*+*^), the stressed SMC cluster (*FOS*^*+*^, *ATF3*^*+*^, *JUN*^*+*^, *HSPB8*^*+*^), two proliferating SMC clusters (*MGP*^*+*^, *TPM4*^*+*^, *MYH10*^*+*^), and the fibromyocyte cluster (*ACTA2*^*+*^, *MYL9*^*+*^, *COL1A2*^*+*^, *COL8A1*^*+*^)^10^. Interestingly, two proliferating SMC clusters exhibited increased expression of synthetic SMC markers and expressed high levels of contractile SMC markers^10^. Therefore, plasticity and multiple phenotypic modulation of SMCs are evident; however, further studies are needed to understand how the multiple phenotypic switching of SMCs is associated with changes in the structure and function of the aortic wall and contributes to disease progression in humans.

In the pathophysiology of aortic aneurysms, immune cell accumulation and activation in aneurysmal lesions are the main features associated with inflammation and structural remodeling of the aortic wall^17^. Single-cell transcriptome analysis showed a heterogeneous monocyte/macrophage/DC distribution in human ascending aortic tissue samples^10^. Macrophage subpopulations were composed of 8 clusters, including M1-like clusters (*TNF*^*+*^, *IL1B*^*+*^, *NFKB1*^*+*^) and M2-like clusters (*MERTK*^*+*^, *MRC1*^*+*^, *STAB1*^*+*^, *CD163*^*+*^)^10^. The M1-like clusters consisted of a few subtypes of macrophages expressing the genes involved in inflammatory function, tissue remodeling, and antigen presentation^10^. In addition, Li et al. identified two M2-like clusters, the M_IFNresponse cluster, the M_remodeling cluster, and the M_proliferating cluster^10^. Another single-cell transcription profiling study on human aortic tissues revealed three monocyte clusters (*CD14*^*+*^, *FCGR3A*^*+*^, *CD36*^*+*^, *HLA-DRA*^*+*^) and macrophage clusters (*CD14*^*+*^, *FCGR3A*^*+*^, *CD68*^*+*^, *TFRC*^*+*^), indicating the upregulation of inflammatory genes involved in cytokine-mediated signaling, nuclear factor-κB transcription factor activity, antigen processing, and T lymphocyte costimulation in monocyte/macrophage populations from AAA samples^18^. In the CaCl_2_-induced AAA mouse model, macrophages were composed of 3 populations: Mφ-1 (*Pf4*^*+*^, *Mrc1*^*+*^), Mφ-2 (*Il1b*^*+*^, *H2-Ab1*^*+*^), and Mφ-3 (*Mki67*^*+*^)^19^. Mφ-1 cells exhibited the gene profile of anti-inflammatory macrophages and were enriched in genes involved in phagocytosis/efferocytosis, including the lysosome, focal adhesions, and endocytosis^19^. Mφ-2 cells had upregulated inflammatory and ECM-degradation pathways, and Mφ-3 cells highly expressed the gene profile of proliferation pathways^19^. Compared with the sham group, the Mφ-2 population was expanded only in the AAA group (2.6% vs. 10.2% in total aortic cells)^19^. In addition, single-cell RNA sequencing revealed five clusters of macrophages (Mo/Mφ_1-5; *Cd14*^*+*^, *Cd68*^*+*^, *Adgre1*^*+*^, *H2-Aa*^*+*^, *Fcgr1*^*+*^) in sham and elastase-treated infrarenal abdominal aorta cells^11^. Consistent with the new concepts of the traditional markers of M1 and M2 states^20,21^, the reparative macrophage cluster Mo/Mφ_3 (*Arg1*^*+*^, *Egr2*^*+*^, *Il1r2*^*+*^) also expressed proinflammatory genes, and the inflammatory macrophage cluster Mo/Mφ_4 (*Ccl2*^*+*^, *Ccl3*^*+*^, *Cxcl10*^*+*^, *Il1b*^*+*^, *Mmp9*^*+*^, *Ctsc*^*+*^) expressed M2 markers^11^. T lymphocytes are also abundant and heterogeneous in the aortic wall^8^, and Li et al. showed that T lymphocytes were the largest cell population in ascending aortic tissues^10^. T lymphocyte subclusters included active CD4 T lymphocytes (*CREM*^*+*^, *CXCR6*^*+*^, *RGCC*^*+*^, *MR3C1*^*+*^, *GZMB*^*+*^), resting CD4 T lymphocytes (*CCR7*^*+*^, *IL7R*^*+*^, *CCL20*^*+*^, *KLRB1*^*+*^), regulatory CD4 T lymphocytes (*IL2RA*^*+*^, *CTLA4*^*+*^, *TNFRSF18*^*+*^, *ID3*^*+*^, *LTB*^*+*^), active CD8 T lymphocytes (*GZMK*^*+*^, *CRTAM*^*+*^, *CCL4*^*+*^, *CMC1*^*+*^), and others (CD8_TEMRA, T_HSP, T_GIMAP, T_stress, T_proliferation, T_switched cluster)^10^. Although various subpopulations of T lymphocytes were identified in aortic tissues, some subpopulations, such as the T_stress and T_HSP clusters, express tissue dissociation-induced genes and may not truly represent the diverse immune cell population associated with aortic aneurysm^10^. Therefore, further studies are needed to verify the roles of the different immune cell populations during the progression of aortic aneurysms.

## Disruption of components of elastin-SMC contractile units in aortic aneurysms

The loss of structural integrity due to vascular SMC dysfunction, including apoptosis and ECM degradation, leads to weakness and dilatation of the aortic wall, which are hallmarks of aortic aneurysm^22^. In healthy vessels, ability of SMCs to contract and relax maintains vascular tone and controls blood pressure and flow; however, under pathological conditions, SMCs switch to a proliferative, synthetic, migratory phenotype that produces ECM to repair vascular injury^3,23^. An imbalance in reparative/ECM production and inflammatory/ECM degradation in SMCs that underwent phenotypic switching and are known as synthetic SMCs in response to constant pathological stimuli damages the aortic wall, leading to dilatation and rupture in aortic aneurysms^24^. Synthetic SMCs have decreased expression of contractile proteins, including α-smooth muscle actin, SM-specific myosin heavy chain, smooth muscle 22α, and SM-calponin, and there is increased production of proteolytic enzymes and ROS to enhance ECM degradation and local inflammation^16,25,26^.

The elastin-SMC contractile unit in the aorta is a functional and structural element that responds to pulsatile blood pressure and flow; thus, mutations in genes involved in the integrity of the ECM and vascular SMC contraction have been identified in the majority of the heritable risk factors for thoracic aortic diseases^6^. Marfan syndrome (MFS) is caused by mutations in the gene encoding fibrillin-1 (*FBN1*), a microfibrillar protein that decorates the surface of elastin fibers, and is characterized by highly penetrant aortic root aneurysms with symptoms in the skeletal and ocular systems^27^. Mutations in *FBN1* induced by missense, frameshift, nonsense, splicing errors, or complete deletion have been identified in patients with MFS, and these patients have decreased fibrillin-1 incorporation into the ECM, which disrupts disulfide pairing and proper folding of the proteins, decreases fibrillin-1 secretion and assembly into microfibrils, or increases the susceptibility to proteolysis^6,28,29^. Therefore, *FBN1* mutations reduce fibrillin-1-containing microfibrils in the aorta, impairing the structural attachment of elastin fibers to SMCs in the medial layer of the aorta^28,29^. In addition to mutations in *FBN1*, mutations in the genes *LOX* and *COL3A1* lead to heritable thoracic aortic disease^30,31^. Lysyl oxidase encoded by *LOX* is responsible for the cross-linking of collagen and elastin, which increases the stabilization of collagen fibrils and the integrity of mature elastin in the ECM^32^. Mutations in *LOX* in patients result in enlargement and dissection of the aortic root and ascending thoracic aorta^30,33^. Mutations in *COL3A1*, which encodes the type III procollagen, are associated with patients with vascular Ehlers‒Danlos syndrome (vEDS), who exhibit a high risk of developing aneurysm, dissection, and rupture of arteries^31^.

The disruption of intracellular components involved in SMC contractile function also causes heritable thoracic aortic diseases^6^. Mutations in genes including *ACTA2*, *MYH11*, and *MYLK* increase the risk of aortic enlargement, aneurysms, or dissections^34–37^. *ACTA2* encodes the SMC-specific isoform of α-actin, which polymerizes to form the thin filaments of the SMC contractile unit, and *MYH11* encodes the SMC-specific myosin heavy chain, which is a major contractile protein^38^. *ACTA2* and *MYH11* gene mutations account for ~10–14% and 2% of familial thoracic aortic diseases, respectively^38,39^. Myosin light chain kinase, which is encoded by *MYLK*, is a ubiquitously expressed kinase that phosphorylates the regulatory light chain of myosin II, leading to SMC contraction^36,37^. Patients with mutations in the *MYLK* gene exhibit disrupted kinase activity and aortic dissection^36,37^. Protein arginine methyltransferase 1 (Prmt1) is a major enzyme associated with the asymmetric arginine demethylation of proteins that are sources of asymmetric dimethylarginine, an endogenous nitric oxide synthase inhibitor^40^. In a recent study, Prmt1 ablation in the aortas of mice impaired SMC contraction and downregulated myocardin expression, inducing a phenotypic switch from contractile to synthetic SMCs^40^. Mice lacking Prmt1 in SMCs exhibited aortic dissection with elastic fiber degeneration and cell death^40^.

## The complexity of the TGF-β signaling pathway in aortic aneurysms

The components of the transforming growth factor-β (TGF-β) signaling pathway, including receptors and SMAD proteins, are fundamental for synthesizing SMC contractile proteins, ECM proteins, elastin, and collagen^41^. Multiple steps are involved in the TGF-β signaling pathway, such as TGF-β synthesis, extracellular deposition, activation of latent TGF-β, direct association with receptors, and initiation of the signal transduction cascade^41^. TGF-β (TGF-β1, 2, and 3) is transcribed from the latency-associated pro-protein, and the removal of the short N-terminal signal peptide allows protein folding and dimerization via disulfide bonds in the endoplasmic reticulum (ER), forming the dimerized pro-TGF-β (the small latent complex, SLC)^42,43^. Cross-linking of the dimerized pro-TGF-β with latent TGF-β binding proteins (LTBPs), which is referred to as the large latent complex (LLC), results in translocation from the ER to the Golgi apparatus^41^. After proteolytic cleavage by furin family proteases, cleaved LLC accumulates in secretory vesicles and is secreted to the extracellular environment^41^. The LLC is incorporated into the ECM and sequentially activated through the cleavage of fibrillin by elastase, the association of integrin receptors with the TGF-β prodomain, the cleavage of fibronectin by bone morphogenetic protein-1, and the degradation of LTBP by matrix metalloproteinase (MMP)-2^41,44,45^. Mature TGF-β is released and associated with type II and type I receptors and initiates intracellular signal transduction, including the activation of SMAD and non-SMAD molecules^41^.

**Fig. 2:**
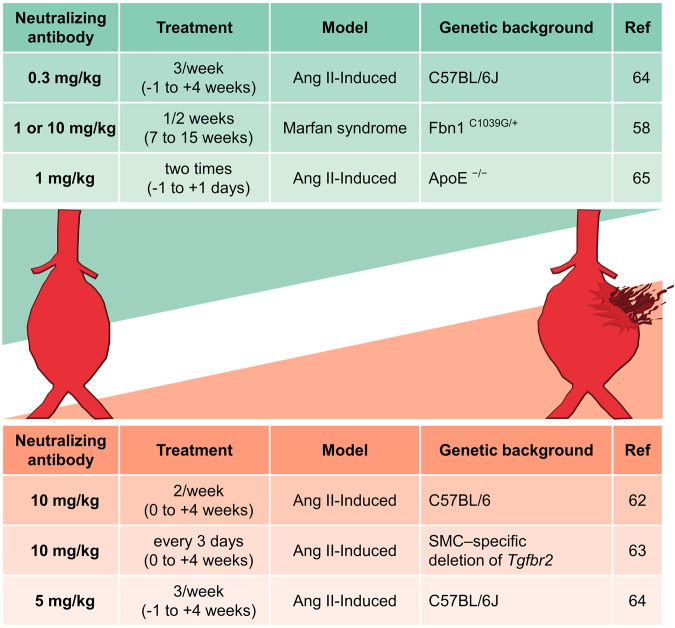
Different consequences of neutralizing TGF-β in mouse aortic aneurysm models. Neutralizing transforming growth factor-β (TGF-β) in mouse aortic aneurysm models results in different effects on the progression of aortic aneurysms depending on the experimental design, including antibody dose, number and timing of the injections, and genetic background. Ang II angiotensin II, ApoE apolipoprotein E, SMC smooth muscle cell.

Although the TGF-β signaling pathway is the primary mechanism for the synthesis of contractile and ECM proteins, the complexity of this signaling means that the intrinsic role of TGF-β in the pathophysiology of aortic aneurysms is unclear (Fig. 2). *Tgfbr2* ablation in SMCs decreases canonical SMAD signaling and reduces the expression and activity of contractile molecules, leading to the activation of stress-related signaling, such as MAPK signaling^46^. Disruption of *Tgfbr2* in postnatal SMCs caused thickness, dilatation, and dissection in the thoracic aorta of WT and mutant fibrillin-1 mice, indicating that basal TGF-β signaling in SMCs was critical for maintaining postnatal aortic wall homeostasis and preventing aortic disease progression^46^. Chen et al. demonstrated that SMC-specific ablation of TGF-β signaling in hypercholesterolemic mice promoted SMC remodeling into MSC-like cells that differentiated osteoblasts, chondrocytes, adipocytes, and macrophages, leading to the development of aortic aneurysms^47^. SMC reprogramming caused by the combination of SMC-specific ablation of TGF-β signaling and hypercholesterolemia resulted in the transdifferentiation of a few medial SMCs to mesenchymal lineage cell types through an increase in Kruppel-like factor 4^47^. Clonal differentiation and expansion of these cells led to the loss of elastin fibers, intramural calcification, high levels of lipid uptake, and severe inflammation, which are all features of human disease^47^. Mutations in the genes involved in the canonical TGF-β signaling pathway, including *TGFBR1*, *TGFBR2*, *SMAD3*, *SMAD4*, and *TGF-β2*, have been identified as predisposing factors for aortic aneurysms and dissections with Marfanoid features^48–52^. Mutations in these genes are predicted or has been proven to decrease the canonical TGF-β signaling pathway^50,51^. Loeys–Dietz syndrome (LDS) is another autosomal dominant genetic connective tissue disorder similar to MFS and vEDS^53^. LDS is caused by mutations in *TGFBR1*, *TGFBR2*, *SMAD3*, *TGF-β2*, and *TGF-β3*, and mutations in these genes cause the production of proteins without functions, leading to a significant reduction in TGF-β signaling output^51,53–55^. Although the mutant receptors lose the capability to transduce TGF-β signaling, tissues from patients and mice with LDS paradoxically show enhanced TGF-β signaling in vivo^56,57^. Excessive TGF-β activity is also observed in the media of aortic aneurysms in patients with MFS, and TGF-β overactivity has been identified in mouse models of MFS caused by deletion or mutation of Fbn1^58–60^. In addition, loss-of-function mutations in the TGF-β repressor SKI, which cause Shprintzen-Goldberg syndrome, increased TGF-β signaling and aortic root aneurysm^61^.

Systemic neutralization of TGF-β activity markedly increased susceptibility to Ang II-induced AAA formation and increased MMP-12 activity in normocholesterolemic C57BL/6 mice^62^. Administration of anti-TGF-β antibodies (10 mg/kg, 2/week) to Ang II-infused C57BL/6 mice led to the development of AAA in 80% of the mice and 40% mortality from aneurysm rupture, and it abrogated serum TGF-β levels and the phosphorylation of SMAD-2 within the aortic wall^62^. Interestingly, Angelov et al. suggested that TGF-β signaling prevented both abdominal and thoracic aneurysms mediated by SMC in extrinsic and intrinsic manners, respectively^63^. Systemic TGF-β neutralization by antibodies (10 mg/kg, every 3 days) increased the prevalence of AAA and increased AAA severity, adventitial thickening, and macrophage accumulation in the aortic wall^63^. SMC-specific loss of *Tgfbr2* accelerated thoracic aortic pathology, including intramural hematomas, medial thinning and adventitial thickening^63^. Another study showed that the administration of anti-TGF-β antibodies (5 mg/kg, 3/week, -1 to +4 weeks) to Ang II-infused C57BL/6 mice reduced serum TGF-β concentrations up to 81% and increased dilatation on ascending and suprarenal aortas and aortic rupture^64^. However, different experimental designs, including the administration of low-dose anti-TGF-β antibodies (0.3 mg/kg, 3/week, -1 to +4 weeks) or delayed treatment of high-dose anti-TGF-β antibodies (5 mg/kg, 3/week, +4 to +8 weeks) to Ang II-infused C57BL/6 mice, had no effect on aortic aneurysm or rupture in mice, even though serum TGF-β concentrations were reduced up to ~40% and 80%, respectively^64^. Notably, Habashi et al. reported that treatment with a TGF-β neutralizing antibody (1 or 10 mg/kg, 1/2 weeks, 7 to 15 weeks of age) prevented aortic aneurism in Fbn1^C1039G/+^ mice, which have the most common class of mutation that causes MFS^58^. A lack of CXCL10, an interferon-inducible chemokine, in Ang II-infused apolipoprotein E (ApoE) knockout mice enriched TGF-β signaling, and neutralizing TGF-β (1 mg/kg, two times) in ApoE ^–/ –^/Cxcl10 ^–/ –^ mice decreased Ang II-induced aortic dilation^65^. Therefore, inhibiting TGF-β signaling to less than physiological levels results in adverse outcomes because it maintains homeostasis in the aortic wall, providing insight for the development of therapeutic agents to limit excessive TGF-β signaling in aortic aneurysms.

## Vascular inflammation in aortic aneurysms

Vascular inflammation is the main initiating factor in aortic aneurysms and substantially influences aortic wall remodeling through the death of aortic wall cells, SMC phenotypic switching, and the secretion of proteases^1^. Innate and adaptive immune cells are related to aortic aneurysms, as indicated by the infiltration of mast cells, macrophages, neutrophils, dendritic cells, B cells, and T cells^10^. Infiltrated immune cells contribute to inflammation in the aortic wall through the secretion of chemokines/cytokines/ROS and then stimulate SMCs to produce various proteases, leading to structural remodeling of the aortic wall^66^.

Chemokines and their receptors initiate a series of inflammatory reactions in aortic aneurysms. CXCR4, a subclass of chemokine receptors, contributed to AAA formation, and its blockade by AMD3100, a potent CXCR4 antagonist, inhibited AAA expansion by reducing the infiltration of adventitial macrophages and aortic wall destruction in a mouse model^67^. Interferon (IFN)-γ and CXCR3 ligands were increased in the plasma of patients with TAA, and CXCR3 knockout in mice revealed protective effects against aneurysm formation with decreased the infiltration of CD45^+^ leukocytes into the aortic wall^68^. Neutralizing CXCR2 with an anti-CXCR2 antibody in acute aortic dissected mice reduced neutrophil accumulation in the tunica adventitia and decreased the levels of local and systemic IL-6, leading to a reduction in aortic rupture^69^. Circulating and aortic C-C chemokine receptor type (CCR) 2^+^ monocytes were increased and positively correlated with suprarenal aortic diameter during Ang II infusion in ApoE-deficient mice^70^. Mast cell migration to AAA lesions in Ang II-infused ApoE-deficient mice promoted AAA formation via CCR2^71^. C-C motif chemokine ligand (CCL) 3, CCR5, and MMP-9 expression was detected in human AAA samples, and intra-aortic CCL3 expression was enhanced in CaCl_2_-induced AAA mouse models^72^. Notably, the loss of CCL3 and CCR5 in mouse models exaggerated AAA, and CCL3 treatment prevented AAA formation in mice by suppressing MMP-9 expression in macrophages^72^.

Cytokines are crucial contributors to inflammatory alterations during AAA formation, and altered expression and epigenetic changes in cytokines were present in AAA tissue samples^73^. After the infiltration of immune cells in the aortic walls, the enhanced production of cytokines stimulates the activation of proteases and induces apoptosis in SMCs, leading to aortic remodeling and rupture^1^. Cytokine profiles revealed 21 of 200 proteins, including cytokines and cytokine receptors, that were differentially expressed in the AAA tissues of Ang II-infused ApoE-deficient mice^74^. Function and pathway enrichment analysis revealed that the differentially expressed proteins were related to leukocyte migration and cell adhesion^74^. In addition, the overexpression and activation of proinflammatory transcription factors upregulated cytokines in AAA^73,75^. Cytokine profiles in homogenized human aortic tissues showed the upregulation of cytokines, including interleukin (IL)-6, IL-1α, IL-1β, tumor necrosis factor (TNF)-α, TNF-β, and oncostatin M^76^.

IL-6, which is a pleiotropic cytokine, is involved in the pathogenesis of various cardiovascular diseases, such as AAA. Ultrasound evaluation of the abdominal aorta revealed the association of circulating levels of IL-6 with abdominal aortic diameter in subjects^77^. Moreover, IL-6 was expressed in the tissue of patients with AAA but not in the corresponding tissue in the control group^78^. Meta-analysis demonstrated that patients with AAA had higher levels of IL-6, and there was an association between a common nonsynonymous functional variant (Asp358Ala; rs2228145) in the IL-6R gene and AAA^79^. Neutralizing the IL-6 receptor in a CaCl_2_-induced AAA mouse model reduced the development of AAA by suppressing Stat3 activity^80^. Notably, selective neutralization of the IL-6 trans-signaling pathway by a soluble form of gp130-Fc but not in both the classical and trans-signaling pathways, improved the survival rate in an AAA mouse model^81^. IL-6 is involved in distinct physiopathological processes, such as anti-inflammatory and proinflammatory responses, which are discriminated by the cascades of the classic and trans-signaling pathways, respectively^82^. IL-1 and TNF-α are key proinflammatory cytokines that regulate and initiate inflammatory responses^73^. Increased levels of IL-1β and TNF-α have been found in the tissue of patients with AAA, and increases in IL-1α and IL-1β plasma levels were detected in patients with AAA^83,84^. However, depending on the pathological conditions, IL-1 and TNF-α may play different roles. Inhibiting IL-1β and TNF-α with antibodies or genetic deletion revealed significantly protected against AAA formation in mouse models^85,86^. In contrast, other studies demonstrated that only inhibiting TNF-α but not IL-1 prevented aortic dilatation in AAA animal models^87,88^. Therefore, further studies are needed to clarify the roles of the IL-1 signaling pathway in aortic aneurysms.

Alterations in the innate immune system, including the upregulation of Toll-like receptors (TLRs), are involved in the pathological process of aortic aneurysm^66,73^. TLRs, which are a transmembrane subtype of pattern recognition receptors, play a critical role in inflammatory responses and innate immunity processes, including the pathological mechanism of aortic aneurysms^89^. Inflammatory cells, ECs and SMCs express TLRs and contribute to inflammatory reactions during aortic aneurysms^66^. The upregulation of TLR2 and its ligands was identified in human AAA tissue, and antagonism of TLR2 in a mouse model decreased the formation and progression of AAA and inhibited chronic inflammation and vascular remodeling^90^. However, whether other TLRs, such as TLR3 and TLR4, are involved in the pathological process of aortic aneurysms, requires further examination^66^. Recent evidence has indicated that the cyclic GMP-AMP synthase (cGAS)-stimulator of interferon genes (STING) pathway, which senses cytosolic DNA, is critical in vascular inflammation and destruction by stimulating innate immune responses^91^. Cytosolic DNA in SMCs and macrophages activate the STING pathway in human sporadic aortic aneurysm and dissection (AAD) tissues^16^. In the sporadic AAD model, DNA leakage into the cytosol activated the STING pathway, inducing death in SMCs, and subsequently delivered DNA into macrophages, where it activated STING and interferon regulatory factor 3, leading to the expression of MMP-9^16^. Thus, cytosolic DNA-mediated activation of the cGAS-STING pathway plays a critical role in aortic degeneration and is a potential therapeutic target for treating aneurysms.

Single-cell transcriptome analysis of aneurysmal human aortic tissues showed that T lymphocytes were the largest cell population^10^, and clonal expansion of infiltrated T lymphocytes was indicated in the aneurysmal aortic wall^9^. During the pathogenesis of AAA, CD4^+^ T cells secrete cytokines, such as Th1 cytokines (IFN-γ, IL-2, and TNF-β) and Th2 cytokines (IL-4, IL-5, IL-6, and IL-10), which are involved in macrophage activation and SMC apoptosis^17^. Depletion of CD4^+^ T cells or IFN-γ in mice prevented aneurysm formation, and reinfusion of IFN-γ in CD4^–/ –^ mice or CD4^+^ T cells in IFN-γ null mice reconstituted aneurysms and orchestrated matrix remodeling^92^. In addition, previous studies demonstrated that aneurysmal tissue expressed Th2 cytokines (IL-4, IL-5, and IL-10) and Th17 cytokines (IL-17)^17^. CD8^+^ T cells were increased in the human AAA wall and promoted cellular apoptosis by releasing IFN-γ and recruiting MMP-producing macrophages in mice^17^.

## Cell death in aortic aneurysms

Cell death and inflammation are closely associated in pathological environments, including aortic aneurysms^93^. Progressive SMC loss is a common pathological feature of aortic aneurysm and dissection. Despite the contribution of SMC phenotype switching and senescence to the loss of SMCs, multiple types of cell death induced by programmed cell death pathways, including apoptosis, necroptosis, ferroptosis, and pyroptosis, are mainly responsible for SMC loss in aneurysmal diseases^93^.

Apoptosis markers, such as fragmented DNA and activated caspase-3, were detected in SMCs in human and animal aortic aneurysm tissues, and the expression of apoptosis-related genes was different in AAA and normal aortic tissue in humans^94,95^. Ang II promotes vascular inflammation through macrophage infiltration in the aortic wall, and these cells produce proteolytic enzymes and proapoptotic mediators, such as perforin, FAS and FAS ligand^93^. In Ang II-treated ApoE-deficient mice, macrophage infiltration, caspase-3 activity, and cytoplasmic histone-associated DNA fragments were increased in the suprarenal aortas in response to Rho-kinase activation^95^. Fasudil, an inhibitor of Rho-kinase, attenuated Ang II-induced AAA and aortic wall apoptosis and proteolysis^95^. ER stress triggered by the unfolded protein response (UPR) induces apoptosis and inflammation, which regulate vascular remodeling in aortic aneurysm and dissection^96^. The transcription factor C/EBP homologous protein (CHOP), a specific factor in the UPR, initiates apoptotic events in response to severe or prolonged ER stress conditions^96^. Mice treated with β-aminopropionitrile (BAPN), a lysyl oxidase inhibitor, developed thoracic AAD, as well as inflammation, excessive apoptosis, and ER stress; however, CHOP deficiency in mice reduced SMC apoptosis and inflammation, which protected against thoracic AAD formation and rupture^96^. Oxidative stress induced by ROS triggered apoptosis in inflammatory responses and enhances aortic aneurysm formation^93^. Inducible nitric oxide synthase (iNOS) and NADPH oxidases are the predominant sources of nitric oxide (NO) and superoxide anion (O_2_^–^) during inflammatory processes, respectively^97^. Selective inhibition of iNOS and NADPH oxidases reduced aneurysm formation by decreasing the production of NO metabolites and the expression of MMP-2 and MMP-9^97^. Peroxiredoxin 2 (Prdx2), a ubiquitous family of thiol-specific antioxidant enzymes that control intracellular peroxide levels, regulates oxidative stress and signal transduction^98^. Loss of Prdx2 in an Ang II-induced AAA mouse model increased SMC death and increased oxidative stress and MMP-2 expression, thereby exacerbating abdominal aortic aneurysm^98^. The expression of phosphodiesterase (PDE) 4D, a cAMP-specific hydrolyzing enzyme, is upregulated in AAA tissue from humans and Ang II-induced mice. PDE4D promoted apoptosis of SMCs by inhibiting the cAMP-activated protein kinase A axis and the phosphorylation of BCL2, which is an antagonist of cell death. Genetic or pharmacological inhibition of PDE4D reduced SMC apoptosis and AAA development in Ang II-induced mice^99^.

Receptor-interacting serine/threonine-protein kinase 3 (RIP3) is a critical mediator of necroptosis, which is regulated by well-orchestrated signaling networks^100^. Increased expression of RIP3 and an increase in necrosis were detected in tissues from patients with AAA and from porcine pancreatic elastase-treated C57BL/6 mice^100^. Depletion of RIP3 or the transplantation of Rip3^+/ –^ aortae to WT mice demonstrated that Rip3 expression in the arterial wall was the primary cause of aneurysm resistance^100^. Overexpression of RIP3 induced SMC necroptosis, and protein kinase C-delta regulated necroptosis by regulating RIP3 expression^100^. Inflammasomes are cytosolic multiprotein oligomers that promote proteolytic cleavage of the proinflammatory cytokines IL-1β and IL-18 and gasdermin-D (GSDMD)^93^. The N-terminal fragment of GSDMD induces a proinflammatory form of programmed cell death referred to as pyroptosis^93^. MCC950, a potent, selective NLR family pyrin domain containing (NLRP) 3 inflammasome inhibitor, inhibited aortic aneurysm and dissection in WT mice fed a high-fat, high-cholesterol diet and infused with Ang II^101^. MCC950 prevented the upregulation of NLRP3 and caspase-1, aortic cell death, and extracellular matrix destruction by MMP-9^101^. Ferroptosis is a form of programmed cell death characterized by high iron-dependent lipid peroxidation, and ferroptosis-related genes are associated with aortic aneurysm formation and dissection^102^. Neutrophil extracellular traps (NETs) promote AAA formation by inducing ferroptosis in SMCs by inhibiting the phosphoinositide 3-kinase (PI3K)/protein kinase B (AKT) pathway, and ferrostatin-1, an inhibitor of ferroptosis, prevents AAA formation^103^.

## Intramural thrombosis and neovascularization in aortic aneurysms

**Fig. 3:**
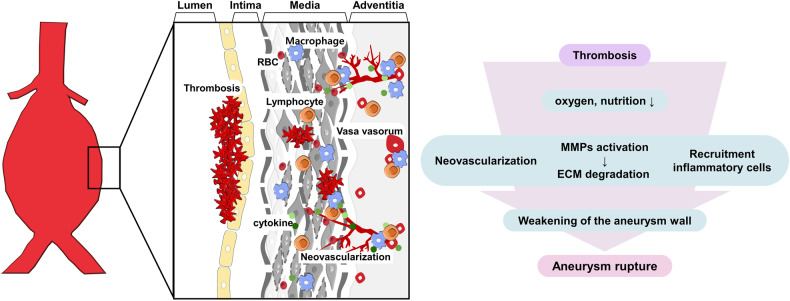
Intramural thrombosis and neovascularization in aortic aneurysm. Thrombus-mediated deprivation of oxygen and nutrients in the aortic wall stimulates neovascularization from the vasa vasorum in the aneurysm wall, inducing the recruitment of inflammatory cells that produce inflammatory mediators and leading to the weakening of the aneurysm wall and aneurysm rupture. RBC red blood cell, MMPs matrix metalloproteinases, ECM extracellular matrix.

Damage to the aortic wall by elastic fiber degradation and deleterious spatial structural remodeling induces coagulation and thrombosis, which results in the maldistribution of oxygen and nutrients from the blood to the aortic wall^104^. Recent studies revealed that intramural thrombus, which is colocalized with the active sites of inflammation and angiogenesis, is closely associated with aortic aneurysm formation^104,105^. Thrombus-mediated deprivation of oxygen and nutrients in the aortic wall stimulates the proliferation of a network of small blood vessels in the aneurysm wall, inducing the recruitment of inflammatory cells that produce inflammatory mediators and leading to the weakening of the aneurysm wall and aneurysm rupture (Fig. 3). During neovascularization, MMP activity is critical for proangiogenic effects, including ECM degradation, EC migration, pericyte detachment from microvessels, angiogenic factor release from the ECM and the cleavage of endothelial junctions^106^.

Vascular endothelial growth factor (VEGF), a major growth factor associated with ECs, promotes EC proliferation, survival, and migration and enhances vascular permeability^107^. The administration of recombinant human VEGF exacerbated the formation of AAAs in Ang II-infused mice and increased the maximum aortic diameter and cross-sectional area of aneurysms, while treatment with VEGF increased MMP-2 gene expression in the aortic wall in Ang II-infused mice^108^. Conversely, inhibiting VEGF-A activity with the soluble VEGF receptor (VEGFR)-2 extracellular ligand-binding domain, an anti-VEGF-A antibody, and the receptor tyrosine kinase inhibitor sunitinib suppressed the enlargement and degradation of AAAs in a mouse model^108,109^. Sequestration of VEGF-A by VERFR-2 attenuated the loss of SMCs, mural angiogenesis, and the infiltration of inflammatory cells, preventing AAA formation^109^. Sunitinib treatment reduced the expression of MMP-2 and MMP-9 in aortic aneurysms and inhibited the chemotaxis of inflammatory cells induced by VEGF-A^109^. Biopsy samples from the aneurysm rupture edge exhibited increased intramural neovascularization, which consisted of smaller diameter and immature microvessels, and there was enhanced expression of angiogenic genes, such as VEGF, α_V_-integrin, and MCP-1^105^. Moreover, the increased density of immature microvessels, showing the weakness of endothelial junctions and mural cell coverage, was identified in the external medial layer in human TAA samples, which contained high levels of pro- and antiangiogenic factors, including angiopoietin-1, angiopoietin-2, fibroblast growth factor-1, and thrombospondin-1^110^.

## Intercellular communication by extracellular vesicles in aortic aneurysms

**Fig. 4:**
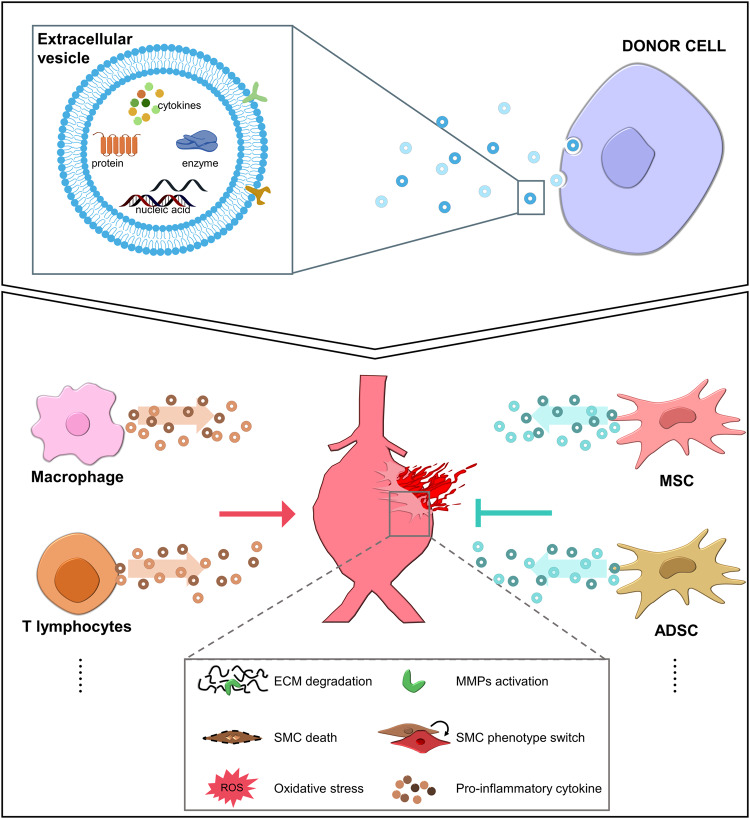
Intercellular communication by extracellular vesicles in aortic aneurysm. Extracellular vesicles (EVs) contain various contents with biological activity, including proinflammatory and anti-inflammatory cytokines, nucleic acids, enzymes, and proteins. EVs are released from donor cells to the bloodstream and are delivered to target cells or organs. Depending on the cargo in EVs, EVs can promote or prevent the progression of aortic aneurysms. MSC mesenchymal stem cell, ADSC adipose-derived mesenchymal stem cell, ECM extracellular matrix, MMPs matrix metalloproteinases, SMC smooth muscle cell, ROS reactive oxygen species.

Extracellular vesicles (EVs), including plasma membrane-derived microvesicles/ectosomes and apoptotic bodies, and endosome-derived exosomes, are pivotal in regulating cell-to-cell communication^111^. Many molecular contents with biological activity, including proinflammatory and anti-inflammatory cytokines, nucleic acids (DNA, RNA, mRNA, microRNA), enzymes, and proteins, are encapsulated in EVs during EV biogenesis^111^. Moreover, EVs have been considered to be crucial mediators of intercellular communication during the pathological course of vascular diseases, including aortic aneurysms (Fig. 4).

Exosomes were abundantly identified in the macrophages of aneurysmal tissues from humans and mice^112^. The administration of GW4869, an inhibitor of exosome biogenesis, significantly attenuated the progression of AAA by reducing elastin degradation and MMP-2 expression in CaPO_4_-induced mice^112^. Exosomes derived from macrophages treated with TNF-α were delivered to SMCs and upregulated the production of MMP-2 in SMCs through the c-Jun N-terminal kinase and p38 pathways^112^. Pyruvate kinase muscle isozyme 2 (PKM2)-activated T lymphocytes produced EVs that were delivered to macrophages, leading to iron accumulation, lipid peroxidation, and migration^113^. T lymphocyte-specific PKM2-null mice or the administration of GW4869 prevented aortic aneurysm formation and decreased aortic diameter, AAA incidence, elastic fiber disruption, MMP expression, and macrophage infiltration^113^. EVs derived from PKM2-activated T lymphocytes contained high levels of polyunsaturated fatty acid-containing phospholipids, providing substrates for lipid peroxidation in macrophages^113^. In addition, increased PKM2 expression was detected in T lymphocytes from AAA subjects, and EVs from the plasma of AAA patients increased iron accumulation, lipid peroxidation, and migration in macrophages^113^.

EVs have been considered an intriguing source of biomarkers to reflect the pathological status of diseases^111^. Human plasma-derived EVs from AAA patients and control subjects exhibited differential protein profiles, and some proteins, such as ferritin, mitochondrial Hsp60, c-reactive protein, and platelet factor 4, which are involved in the main pathological mechanism of AAA, including oxidative stress, inflammation, and thrombosis, were detected in EVs in the plasma of AAA patients^114^. In addition, ficolin-3, a molecule in the lectin complement-activation pathway, was involved in AAA pathophysiology and was increased in EVs obtained from activated platelets and AAA tissue^115^. Increased plasma ficolin-3 levels were positively associated with aortic diameter, indicating its potential as a potent biomarker for aneurysmal growth^115^. MicroRNAs (miRNAs) are small single-stranded noncoding RNA molecules with a length of ~22 nucleotides, and miRNA levels in EVs differ depending on specific environments^111^. EVs in the serum of patients with AAA exhibited differences in miR-122-5p, miR-193a-5p, miR-543, miR-576-3p, miR-629-5p, miR-2110, and miR-483-5p, which regulate biological functions involved in cell growth, aging, neuron death, vasculature development, kinase signaling pathway, and the TGF-β response^116^.

The therapeutic potential of EVs derived from various stem cells has been suggested to have multiple benefits, including biological activity, transmission efficiency, stability, and safety. The administration of MSC-derived EVs containing miR-147 attenuated aortic diameter, proinflammatory cytokine levels, inflammatory cell infiltration, and elastic fiber disruption and increased the expression of smooth muscle cell α-actin in elastase-treated mice^117^. NETs promoted AAA formation by inducing SMC ferroptosis by inhibiting the PI3K/AKT pathway^103^. MSC-EVs redirected NETosis to apoptosis in neutrophils, inhibiting NET release and maintaining the PI3K/AKT pathway, thereby preventing AAA formation in an Ang II-induced AAA mouse model^103^. Exosomal miR-17-5p derived from adipose-derived mesenchymal stem cells (ADSCs) decreased thioredoxin-interacting protein-induced pyroptosis in macrophages induced by Ang II, and the overexpression of miR-17-5p enhanced the therapeutic potential of ADSC-derived exosomes in an AAA mouse model^118^.

## Mouse models of aortic aneurysm

Mouse models have been developed to simulate the characteristics of dissecting and nondissecting aortic aneurysms in humans (Table 2). These models aim to mimic the diverse patterns of location and structure, the existence or nonexistence of a blood clot and gradual growth compared with abrupt rupture^119^.

**Table 2:** Mouse models used for aortic aneurysm studies.

Model	Surgery	Dissection	Rupture	ECM degradation	Leukocyte	IMT	ILT
Human Pathology	**–**	**+**	**+**	**+**	**+**	**+**	**+**
Ang II infusion	Minor	**+**	**+**	**+**	**+**	**+**	**–**
CaCl_2_	Major	**–**	**–**	**+**	**+**	**–**	**–**
Elastase	Major	**–**	**+**	**+**	**+**	**–**	**+**
MCR Agonist + Salt	Major	**+**	**+**	**+**	**+**	**+**	**–**
BAPN	Minor	**+**	**+**	**+**	**–**	**–**	**–**
Xenograft	Major	**–**	**–**	**+**	**+**	**–**	**+**
Saccular	Major	**+**	**+**	**+**	**+**	**–**	**+**

*Ang II* angiotensin II, *CaCl*_*2*_ calcium chloride, *MCR* mineralocorticoid receptor, *BAPN* β-aminopropionitrile, *ECM* extracellular matrix, *IMT* intramural thrombosis, *ILT* intraluminal thrombosis.

Aneurysms with dissections have a prevalence ranging from 1.3% to 8%. These conditions are closely linked to a substantial mortality risk due to acute complications in the aorta^120^. Aortic aneurysm and dissection are characterized by contained intramural rupture, which leads to the entry of blood into the space between the inner and middle layers of the aorta, forming a hematoma within the wall itself^1^. Methods used to develop dissecting AAA models include the perfusion of Ang II, the administration of mineralocorticoid receptor agonists, high salt administration and inactivation of the *Lox* gene using BAPN^4,121^.

Nondissecting aneurysms are distinguished by the absence of separation or tearing within the layers of the arterial wall, and they do not involve the splitting or detachment of arterial wall layers^121^. These models closely mimic human pathology, including the infiltration of inflammatory cells, the loss of vascular SMCs and MMP-mediated ECM degradation^121^. However, this model is mild, and it does not exhibit two prominent characteristics often found in human AAA: the presence of a thrombus and aortic rupture^121^. Researchers have used various approaches to study nondissecting AAA models, including the elastase-induced model, decellularized aortic xenograft model, saccular aneurysm model, and the calcium chloride adventitial application model^4,121^. Nondissecting aneurysms typically manifest within 2 weeks, making this model appropriate for preventive studies^121^.

Animal models of TAA are constructed by mutating specific genes involved in the degradation of ECM, contractile dysfunction of SMCs, and dysfunction of the TGF-β signaling pathway because genetic factors are key for the development of TAA^4^. Table 3 shows mouse models used to study human genetic disorders that are associated with aortic aneurysms. Among various mouse models used to study AAA, the three most frequently used methods involve continuous subcutaneous administration of Ang II, exposing the adventitia to CaCl_2_ and temporally infusing elastase into the infrarenal aorta^121^.

**Table 3:** Mouse models of human genetic disorders involving aortic aneurysms.

Gene	Mouse model	Disorder
Fbn1	Fbn1 ^tm1Hcd^	Marfan syndrome (MFS)
	Fbn1 ^tm2Rmz^	
Col3a1	Col3a1 ^em1Hcd^	Vascular Ehlers-Danlos syndrome (EDS)
Col5a1	Col5a1 ^+/–^	
Col5a2	Col5a2 ^floxed/floxed^	
Tgfbr1	Tgfbr1 ^tm1.1Hcd^	Loeys‒Dietz syndrome (LDS)
	Tgfbr1 ^M318R/+^	
Tgfbr2	Tgfbr2 ^tm1.1Hcd^	
	Tgfbr2 ^G357W/+^	
Tgfb2	Tgfb2 ^+/–^	
Smad3	Smad3 ^SmKO^	
Lox1	Lox ^em1Mech^	Familial thoracic aortic aneurysm and aortic dissection (Familial TAAD)
Myh11	Myh11 ^∆K/∆K^	
	Myh11 ^R247C/R247C^	
Mylk	Mylk ^SMKO^	
Acta2	Acta2 ^–/–^	
Fbln4	Fbln4 ^SMKO^	
Fbln5	Fbln5 ^–/–^	

Daugherty et al. first reported in 2000 that chronic administration of Ang II (500 or 1000 ng/kg/min) for 28 days using a minipump implanted in 6-month-old ApoE-deficient mice resulted in a distended abdominal aortic shape in 20% and 33% of the mice^122,123^. In the case of low-density lipoprotein receptor-deficient mice, they also present a similar extent for AAA studies^124^. The pharmacological model of continuous Ang II infusion offers the benefit of a simple surgical procedure, eliminating the need for invasive aortic manipulations^123^. Ang II infusion results in characteristics such as macrophage infiltration, degradation of the elastic media, the occurrence of aortic dissection, the formation of intramural thrombus, remodeling of the aneurysmal wall and eventual aortic rupture^125^.

Periaortic administration of CaCl_2_ onto the infrarenal aorta to induce AAA was initially described in rabbits as an animal model of AAA^126,127^. Subsequently, this technique was modified and used in mice for further research purposes^128,129^. One of the advantages of this model is that it can be performed on wild-type mice without any genetic manipulation^121,128^. This model exhibits several characteristics that closely resemble specific aspects of human AAA, including calcification, elastin degradation, programmed cell death in SMCs, the clearance of cellular debris through phagocytosis and increased enzymatic degradation within aortic tissue^128^. The CaCl_2_-induced AAA model exhibits distinct differences from human AAA, including the absence of intraluminal thrombus and aortic rupture, which are commonly observed in the classical presentation of the disease^123^.

In the elastase-induced aneurysm model, porcine pancreatic elastase (PPE) is administered to the infrarenal segment of the aorta by direct infusion or application^121^. The first reported model of elastase-induced AAA involved exposing a specific section of the aorta to elastase through perfusion, as introduced by Anidjar et al. in 1990, in rats^130^. In 2012, Bhamidipati et al. introduced a modified approach in which they performed periadventitial administration of PPE^131^. This method has become preferred for most surgeries to create the elastase-induced AAA model^130,131^. As elastase infiltrates the medial layer, it damages the elastic fibers and initiates arterial dilatation when unclamping occurs^130,131^. Consequently, a gradual process of deterioration of medial elastic fibers results, ultimately culminating in the development of an AAA^130,131^.

## Conclusion

There are no effective medical therapies to prevent the growth or rupture of aortic aneurysms at present, and long-term clinical trials for potential drugs, including Ang II converting enzyme inhibitors, angiotensin receptor blockers, β-blockers, and statins, have shown limited efficacy in controlling aortic aneurysms. However, recent advanced technologies in single-cell analysis, proteomics, and artificial intelligence will provide novel opportunities to identify various targets involved in cellular heterogeneity, SMC phenotypic switching, vascular inflammation, cell death, ECM degradation, intramural thrombosis, and extracellular vesicles. Many target molecules in preclinical findings should be validated to develop efficient drugs to treat patients with aortic aneurysms. Therefore, innovative translational research is necessary to overcome the hurdles that delay validating the relevance and efficacy of preclinical findings to clinical applications.

## References

[1] Golledge J (2019). Abdominal aortic aneurysm: update on pathogenesis and medical treatments. Nat. Rev. Cardiol..

[2] Liu B, Granville DJ, Golledge J, Kassiri Z (2020). Pathogenic mechanisms and the potential of drug therapies for aortic aneurysm. Am. J. Physiol. Heart Circ. Physiol..

[3] Qian, G., Adeyanju, O., Olajuyin, A. & Guo, X. Abdominal aortic aneurysm formation with a focus on vascular smooth muscle cells. *Life* **12**, 191 (2022). 10.3390/life12020191 PMC8880357 35207478

[4] Gao J (2023). The mechanism and therapy of aortic aneurysms. Signal Transduct. Target. Ther..

[5] Shen YH, LeMaire SA (2017). Molecular pathogenesis of genetic and sporadic aortic aneurysms and dissections. Curr. Probl. Surg..

[6] Pinard A, Jones GT, Milewicz DM (2019). Genetics of thoracic and abdominal aortic diseases. Circ. Res..

[7] Kuivaniemi H, Ryer EJ, Elmore JR, Tromp G (2015). Understanding the pathogenesis of abdominal aortic aneurysms. Expert Rev. Cardiovasc. Ther..

[8] Li Y, LeMaire SA, Shen YH (2021). Molecular and cellular dynamics of aortic aneurysms revealed by single-cell transcriptomics. Arterioscler. Thromb. Vasc. Biol..

[9] Jauhiainen S, Kiema M, Hedman M, Laakkonen JP (2022). Large vessel cell heterogeneity and plasticity: focus in aortic aneurysms. Arterioscler. Thromb. Vasc. Biol..

[10] Li Y (2020). Single-cell transcriptome analysis reveals dynamic cell populations and differential gene expression patterns in control and aneurysmal human aortic tissue. Circulation.

[11] Zhao G (2021). Single-cell RNA sequencing reveals the cellular heterogeneity of aneurysmal infrarenal abdominal aorta. Cardiovasc. Res..

[12] Branchetti E (2013). Oxidative stress modulates vascular smooth muscle cell phenotype via CTGF in thoracic aortic aneurysm. Cardiovasc. Res..

[13] Fei J (2016). Novel mechanism controlling phenotypic modulation of vascular smooth muscle cells. Circ. Res..

[14] Liu R (2013). Ten-eleven translocation-2 (TET2) is a master regulator of smooth muscle cell plasticity. Circulation.

[15] Dobnikar L (2018). Disease-relevant transcriptional signatures identified in individual smooth muscle cells from healthy mouse vessels. Nat. Commun..

[16] Luo W (2020). Critical role of cytosolic DNA and its sensing adaptor STING in aortic degeneration, dissection, and rupture. Circulation.

[17] Yuan Z (2020). Abdominal aortic aneurysm: roles of inflammatory cells. Front. Immunol..

[18] Davis, F. M. et al. Inhibition of macrophage histone demethylase JMJD3 protects against abdominal aortic aneurysms. *J. Exp. Med*. **218**, e20201839 (2021). 10.1084/jem.20201839 PMC8008365 33779682

[19] Yang H, Zhou T, Stranz A, DeRoo E, Liu B (2021). Single-cell RNA sequencing reveals heterogeneity of vascular cells in early stage murine abdominal aortic aneurysm-brief report. Arterioscler. Thromb. Vasc. Biol..

[20] Mould, K. J., Jackson, N. D., Henson, P. M., Seibold, M. & Janssen, W. J. Single cell RNA sequencing identifies unique inflammatory airspace macrophage subsets. *JCI Insight* **4**, e126556 (2019). 10.1172/jci.insight.126556 PMC6483508 30721157

[21] Nahrendorf M, Swirski FK (2016). Abandoning M1/M2 for a network model of macrophage function. Circ. Res..

[22] Quintana RA, Taylor WR (2019). Cellular mechanisms of aortic aneurysm formation. Circ. Res..

[23] Lu H (2021). Vascular smooth muscle cells in aortic aneurysm: from genetics to mechanisms. J. Am. Heart Assoc..

[24] Petsophonsakul P (2019). Role of vascular smooth muscle cell phenotypic switching and calcification in aortic aneurysm formation. Arterioscler. Thromb. Vasc. Biol..

[25] Hadi T (2018). Macrophage-derived netrin-1 promotes abdominal aortic aneurysm formation by activating MMP3 in vascular smooth muscle cells. Nat. Commun..

[26] McCormick ML, Gavrila D, Weintraub NL (2007). Role of oxidative stress in the pathogenesis of abdominal aortic aneurysms. Arterioscler. Thromb. Vasc. Biol..

[27] Pyeritz RE, McKusick VA (1979). The Marfan syndrome: diagnosis and management. N. Engl. J. Med..

[28] Dietz HC, Saraiva JM, Pyeritz RE, Cutting GR, Francomano CA (1992). Clustering of fibrillin (FBN1) missense mutations in Marfan syndrome patients at cysteine residues in EGF-like domains. Hum. Mutat..

[29] Whiteman P, Hutchinson S, Handford PA (2006). Fibrillin-1 misfolding and disease. Antioxid. Redox Signal..

[30] Lee VS (2016). Loss of function mutation in LOX causes thoracic aortic aneurysm and dissection in humans. Proc. Natl Acad. Sci. USA.

[31] Gdynia HJ, Kuhnlein P, Ludolph AC, Huber R (2008). Connective tissue disorders in dissections of the carotid or vertebral arteries. J. Clin. Neurosci..

[32] Csiszar K (2001). Lysyl oxidases: a novel multifunctional amine oxidase family. Prog. Nucleic Acid Res. Mol. Biol..

[33] Guo DC (2016). LOX mutations predispose to thoracic aortic aneurysms and dissections. Circ. Res..

[34] Guo DC (2009). Mutations in smooth muscle alpha-actin (ACTA2) cause coronary artery disease, stroke, and Moyamoya disease, along with thoracic aortic disease. Am. J. Hum. Genet..

[35] Zhu L (2006). Mutations in myosin heavy chain 11 cause a syndrome associating thoracic aortic aneurysm/aortic dissection and patent ductus arteriosus. Nat. Genet..

[36] Shalata A (2018). Fatal thoracic aortic aneurysm and dissection in a large family with a novel MYLK gene mutation: delineation of the clinical phenotype. Orphanet. J. Rare Dis..

[37] Wallace SE (2019). MYLK pathogenic variants aortic disease presentation, pregnancy risk, and characterization of pathogenic missense variants. Genet. Med..

[38] Guo DC (2007). Mutations in smooth muscle alpha-actin (ACTA2) lead to thoracic aortic aneurysms and dissections. Nat. Genet..

[39] Pannu H (2007). MYH11 mutations result in a distinct vascular pathology driven by insulin-like growth factor 1 and angiotensin II. Hum. Mol. Genet..

[40] Pyun JH (2021). Inducible Prmt1 ablation in adult vascular smooth muscle leads to contractile dysfunction and aortic dissection. Exp. Mol. Med..

[41] Tzavlaki, K. & Moustakas, A. TGF-beta signaling. *Biomolecules* **10**, 487 (2020). 10.3390/biom10030487 PMC7175140 32210029

[42] Derynck, R. & Budi, E. H. Specificity, versatility, and control of TGF-beta family signaling. *Sci. Signal* **12**, eaav5183 (2019). 10.1126/scisignal.aav5183 PMC6800142 30808818

[43] ten Dijke P, Arthur HM (2007). Extracellular control of TGFbeta signalling in vascular development and disease. Nat. Rev. Mol. Cell Biol..

[44] Robertson, I. B. & Rifkin, D. B. Regulation of the bioavailability of TGF-beta and TGF-beta-related proteins. *Cold Spring Harb. Perspect. Biol*. **8**, a021907 (2016). 10.1101/cshperspect.a021907 PMC4888822 27252363

[45] Ge G, Greenspan DS (2006). BMP1 controls TGFbeta1 activation via cleavage of latent TGFbeta-binding. protein J. Cell Biol..

[46] Li W (2014). Tgfbr2 disruption in postnatal smooth muscle impairs aortic wall homeostasis. J. Clin. Invest..

[47] Chen PY (2020). Smooth muscle cell reprogramming in aortic aneurysms. Cell Stem Cell.

[48] Mizuguchi T (2004). Heterozygous TGFBR2 mutations in Marfan syndrome. Nat. Genet..

[49] Tran-Fadulu V (2009). Analysis of multigenerational families with thoracic aortic aneurysms and dissections due to TGFBR1 or TGFBR2. Mutat. J. Med. Genet..

[50] Lindsay ME (2012). Loss-of-function mutations in TGFB2 cause a syndromic presentation of thoracic aortic aneurysm. Nat. Genet..

[51] Boileau C (2012). TGFB2 mutations cause familial thoracic aortic aneurysms and dissections associated with mild systemic features of Marfan syndrome. Nat. Genet..

[52] Schepers D (2018). A mutation update on the LDS-associated genes TGFB2/3 and SMAD2/3. Hum. Mutat..

[53] Loeys BL (2006). Aneurysm syndromes caused by mutations in the TGF-beta receptor. N. Engl. J. Med..

[54] Bertoli-Avella AM (2015). Mutations in a TGF-beta ligand, TGFB3, cause syndromic aortic aneurysms and dissections. J. Am. Coll. Cardiol..

[55] van de Laar IM (2011). Mutations in SMAD3 cause a syndromic form of aortic aneurysms and dissections with early-onset osteoarthritis. Nat. Genet..

[56] Gallo EM (2014). Angiotensin II-dependent TGF-beta signaling contributes to Loeys-Dietz syndrome vascular pathogenesis. J. Clin. Invest..

[57] Hara H (2019). Activation of TGF-beta signaling in an aortic aneurysm in a patient with Loeys-Dietz syndrome caused by a novel loss-of-function variant of TGFBR1. Hum. Genome Var..

[58] Habashi JP (2006). Losartan, an AT1 antagonist, prevents aortic aneurysm in a mouse model of Marfan syndrome. Science.

[59] Neptune ER (2003). Dysregulation of TGF-beta activation contributes to pathogenesis in Marfan syndrome. Nat. Genet..

[60] Ruddy JM (2009). Differential effects of mechanical and biological stimuli on matrix metalloproteinase promoter activation in the thoracic aorta. Circulation.

[61] Doyle AJ (2012). Mutations in the TGF-beta repressor SKI cause Shprintzen-Goldberg syndrome with aortic aneurysm. Nat. Genet..

[62] Wang Y (2010). TGF-beta activity protects against inflammatory aortic aneurysm progression and complications in angiotensin II-infused mice. J. Clin. Invest..

[63] Angelov SN (2017). TGF-beta (Transforming Growth Factor-beta) signaling protects the thoracic and abdominal aorta from angiotensin II-induced pathology by distinct mechanisms. Arterioscler. Thromb. Vasc. Biol..

[64] Chen X (2016). TGF-beta neutralization enhances angII-induced aortic rupture and aneurysm in both thoracic and abdominal regions. PLoS One.

[65] King VL (2009). Interferon-gamma and the interferon-inducible chemokine CXCL10 protect against aneurysm formation and rupture. Circulation.

[66] Li H (2018). Modulation of immune-inflammatory responses in abdominal aortic aneurysm: emerging molecular targets. J. Immunol. Res..

[67] Michineau S (2014). Chemokine (C-X-C motif) receptor 4 blockade by AMD3100 inhibits experimental abdominal aortic aneurysm expansion through anti-inflammatory effects. Arterioscler. Thromb. Vasc. Biol..

[68] Gallo A (2012). Circulating interferon-gamma-inducible Cys-X-Cys chemokine receptor 3 ligands are elevated in humans with aortic aneurysms and Cys-X-Cys chemokine receptor 3 is necessary for aneurysm formation in mice. J. Thorac. Cardiovasc. Surg..

[69] Anzai A (2015). Adventitial CXCL1/G-CSF expression in response to acute aortic dissection triggers local neutrophil recruitment and activation leading to aortic rupture. Circ. Res..

[70] Moran CS (2013). Everolimus limits aortic aneurysm in the apolipoprotein E-deficient mouse by downregulating C-C chemokine receptor 2 positive monocytes. Arterioscler. Thromb. Vasc. Biol..

[71] Zhang J (2012). Chemokine (C-C motif) receptor 2 mediates mast cell migration to abdominal aortic aneurysm lesions in mice. Cardiovasc. Res..

[72] Ishida Y (2020). Prevention of CaCl(2)-induced aortic inflammation and subsequent aneurysm formation by the CCL3-CCR5 axis. Nat. Commun..

[73] Puchenkova OA (2022). Cytokines in abdominal aortic aneurysm: master regulators with clinical application. Biomark. Insights.

[74] Li Y (2019). Discovery of crucial cytokines associated with abdominal aortic aneurysm formation by protein array analysis. Exp. Biol. Med..

[75] Lindeman JH (2008). Enhanced expression and activation of pro-inflammatory transcription factors distinguish aneurysmal from atherosclerotic aorta: IL-6- and IL-8-dominated inflammatory responses prevail in the human aneurysm. Clin. Sci..

[76] Middleton RK (2007). The pro-inflammatory and chemotactic cytokine microenvironment of the abdominal aortic aneurysm wall: a protein array study. J. Vasc. Surg..

[77] Rohde LE (1999). Plasma concentrations of interleukin-6 and abdominal aortic diameter among subjects without aortic dilatation. Arterioscler. Thromb. Vasc. Biol..

[78] Yuwen L (2019). A pilot study of protein microarray for simultaneous analysis of 274 cytokines between abdominal aortic aneurysm and normal aorta. Angiology.

[79] Harrison SC (2013). Interleukin-6 receptor pathways in abdominal aortic aneurysm. Eur. Heart J..

[80] Nishihara M (2017). The role of IL-6 in pathogenesis of abdominal aortic aneurysm in mice. PLoS One.

[81] Paige E (2019). Interleukin-6 receptor signaling and abdominal aortic aneurysm growth rates. Circ. Genom. Precis. Med..

[82] Garbers C, Heink S, Korn T, Rose-John S (2018). Interleukin-6: designing specific therapeutics for a complex cytokine. Nat. Rev. Drug Discov..

[83] Newman KM, Jean-Claude J, Li H, Ramey WG, Tilson MD (1994). Cytokines that activate proteolysis are increased in abdominal aortic aneurysms. Circulation.

[84] Wu X (2016). Sex- and disease-specific inflammasome signatures in circulating blood leukocytes of patients with abdominal aortic aneurysm. Mol. Med..

[85] Xiong W (2009). Blocking TNF-alpha attenuates aneurysm formation in a murine model. J. Immunol..

[86] Isoda K (2018). Inhibition of interleukin-1 suppresses angiotensin II-induced aortic inflammation and aneurysm formation. Int. J. Cardiol..

[87] Hingorani A (1998). The effect of tumor necrosis factor binding protein and interleukin-1 receptor antagonist on the development of abdominal aortic aneurysms in a rat model. J. Vasc. Surg..

[88] Batra R (2018). IL-1beta (Interleukin-1beta) and TNF-alpha (Tumor Necrosis Factor-alpha) impact abdominal aortic aneurysm formation by differential effects on macrophage polarization. Arterioscler. Thromb. Vasc. Biol..

[89] Pryshchep O, Ma-Krupa W, Younge BR, Goronzy JJ, Weyand CM (2008). Vessel-specific Toll-like receptor profiles in human medium and large arteries. Circulation.

[90] Yan H (2015). Antagonism of toll-like receptor 2 attenuates the formation and progression of abdominal aortic aneurysm. Acta Pharm. Sin. B.

[91] Decout A, Katz JD, Venkatraman S, Ablasser A (2021). The cGAS-STING pathway as a therapeutic target in inflammatory diseases. Nat. Rev. Immunol..

[92] Xiong W, Zhao Y, Prall A, Greiner TC, Baxter BT (2004). Key roles of CD4+ T cells and IFN-gamma in the development of abdominal aortic aneurysms in a murine model. J. Immunol..

[93] Chakraborty A (2022). Programmed cell death in aortic aneurysm and dissection: a potential therapeutic target. J. Mol. Cell Cardiol..

[94] Henderson EL (1999). Death of smooth muscle cells and expression of mediators of apoptosis by T lymphocytes in human abdominal aortic aneurysms. Circulation.

[95] Wang YX (2005). Fasudil, a Rho-kinase inhibitor, attenuates angiotensin II-induced abdominal aortic aneurysm in apolipoprotein E-deficient mice by inhibiting apoptosis and proteolysis. Circulation.

[96] Jia LX (2015). Mechanical stretch-induced endoplasmic reticulum stress, apoptosis and inflammation contribute to thoracic aortic aneurysm and dissection. J. Pathol..

[97] Xiong W (2009). Inhibition of reactive oxygen species attenuates aneurysm formation in a murine model. Atherosclerosis.

[98] Jeong SJ (2020). Deficiency of peroxiredoxin 2 exacerbates angiotensin II-induced abdominal aortic aneurysm. Exp. Mol. Med..

[99] Gao R (2022). Phosphodiesterase 4D contributes to angiotensin II-induced abdominal aortic aneurysm through smooth muscle cell apoptosis. Exp. Mol. Med..

[100] Wang Q (2015). Receptor-interacting protein kinase 3 contributes to abdominal aortic aneurysms via smooth muscle cell necrosis and inflammation. Circ. Res..

[101] Ren P (2020). Targeting the NLRP3 inflammasome with inhibitor MCC950 prevents aortic aneurysms and dissections in mice. J. Am. Heart Assoc..

[102] Ren J (2022). Key ferroptosis-related genes in abdominal aortic aneurysm formation and rupture as determined by combining bioinformatics techniques. Front. Cardiovasc. Med..

[103] Chen L (2023). Mesenchymal stem cell-derived extracellular vesicles protect against abdominal aortic aneurysm formation by inhibiting NET-induced ferroptosis. Exp. Mol. Med..

[104] Choke E (2006). Increased angiogenesis at the site of abdominal aortic aneurysm rupture. Ann. N. Y. Acad. Sci..

[105] Choke E (2006). Abdominal aortic aneurysm rupture is associated with increased medial neovascularization and overexpression of proangiogenic cytokines. Arterioscler. Thromb. Vasc. Biol..

[106] Cockerill GW, Gamble JR, Vadas MA (1995). Angiogenesis: models and modulators. Int. Rev. Cytol..

[107] Nagy JA, Dvorak AM, Dvorak HF (2007). VEGF-A and the induction of pathological angiogenesis. Annu. Rev. Pathol..

[108] Choke E (2010). Vascular endothelial growth factor enhances angiotensin II-induced aneurysm formation in apolipoprotein E-deficient mice. J. Vasc. Surg..

[109] Xu B (2019). Inhibition of VEGF (Vascular Endothelial Growth Factor)-A or its receptor activity suppresses experimental aneurysm progression in the aortic elastase infusion model. Arterioscler. Thromb. Vasc. Biol..

[110] Kessler K (2014). Angiogenesis and remodelling in human thoracic aortic aneurysms. Cardiovasc. Res..

[111] Lu S, Wang R, Fu W, Si Y (2022). Applications of extracellular vesicles in abdominal aortic aneurysm. Front. Cardiovasc. Med..

[112] Wang Y (2019). Involvement of macrophage-derived exosomes in abdominal aortic aneurysms development. Atherosclerosis.

[113] Dang G (2022). T lymphocyte-derived extracellular vesicles aggravate abdominal aortic aneurysm by promoting macrophage lipid peroxidation and migration via pyruvate kinase muscle isozyme 2. Redox Biol..

[114] Martinez-Pinna R, Gonzalez de Peredo A, Monsarrat B, Burlet-Schiltz O, Martin-Ventura JL (2014). Label-free quantitative proteomic analysis of human plasma-derived microvesicles to find protein signatures of abdominal aortic aneurysms. Proteom. Clin. Appl..

[115] Fernandez-Garcia CE (2017). Association of ficolin-3 with abdominal aortic aneurysm presence and progression. J. Thromb. Haemost..

[116] Hildebrandt A (2021). Detection of atherosclerosis by small RNA-sequencing analysis of extracellular vesicle enriched serum samples. Front. Cell Dev. Biol..

[117] Spinosa M (2018). Human mesenchymal stromal cell-derived extracellular vesicles attenuate aortic aneurysm formation and macrophage activation via microRNA-147. FASEB J..

[118] Hu J (2022). Exosomal miR-17-5p from adipose-derived mesenchymal stem cells inhibits abdominal aortic aneurysm by suppressing TXNIP-NLRP3 inflammasome. Stem Cell Res. Ther..

[119] Senemaud J (2017). Translational relevance and recent advances of animal models of abdominal aortic aneurysm. Arterioscler. Thromb. Vasc. Biol..

[120] Erbel R (2014). 2014 ESC guidelines on the diagnosis and treatment of aortic diseases: document covering acute and chronic aortic diseases of the thoracic and abdominal aorta of the adult. The task force for the diagnosis and treatment of aortic diseases of the European Society of Cardiology (ESC). Eur. Heart J..

[121] Golledge J, Krishna SM, Wang Y (2022). Mouse models for abdominal aortic aneurysm. Br. J. Pharmacol..

[122] Daugherty A, Manning MW, Cassis LA (2000). Angiotensin II promotes atherosclerotic lesions and aneurysms in apolipoprotein E-deficient. Mice. J. Clin. Invest..

[123] Daugherty A, Cassis LA (2004). Mouse models of abdominal aortic aneurysms. Arterioscler. Thromb. Vasc. Biol..

[124] Daugherty A, Cassis L (1999). Chronic angiotensin II infusion promotes atherogenesis in low density lipoprotein receptor -/- mice. Ann. N. Y. Acad. Sci..

[125] Daugherty A, Rateri DL, Cassis LA (2006). Role of the renin-angiotensin system in the development of abdominal aortic aneurysms in animals and humans. Ann. N. Y. Acad. Sci..

[126] Gertz SD, Kurgan A, Eisenberg D (1988). Aneurysm of the rabbit common carotid artery induced by periarterial application of calcium chloride in vivo. J. Clin. Invest..

[127] Freestone T, Turner RJ, Higman DJ, Lever MJ, Powell JT (1997). Influence of hypercholesterolemia and adventitial inflammation on the development of aortic aneurysm in rabbits. Arterioscler. Thromb. Vasc. Biol..

[128] Chiou AC, Chiu B, Pearce WH (2001). Murine aortic aneurysm produced by periarterial application of calcium chloride. J. Surg. Res..

[129] Longo GM (2002). Matrix metalloproteinases 2 and 9 work in concert to produce aortic aneurysms. J. Clin. Invest..

[130] Anidjar S (1990). Elastase-induced experimental aneurysms in rats. Circulation.

[131] Bhamidipati CM (2012). Development of a novel murine model of aortic aneurysms using peri-adventitial elastase. Surgery.

